# A haplotype-resolved reference genome for *Eucalyptus grandis*

**DOI:** 10.1093/g3journal/jkaf112

**Published:** 2025-05-30

**Authors:** Anneri Lötter, Tomas Bruna, Tuan A Duong, Kerrie Barry, Anna Lipzen, Chris Daum, Yuko Yoshinaga, Jane Grimwood, Jerry W Jenkins, Jayson Talag, Justin Borevitz, John T Lovell, Jeremy Schmutz, Jill L Wegrzyn, Alexander A Myburg

**Affiliations:** Department of Biochemistry, Genetics and Microbiology, Forestry and Agricultural Biotechnology Institute (FABI), University of Pretoria, Private Bag X20, Pretoria 0028, South Africa; Department of Energy Joint Genome Institute, Lawrence Berkeley National Laboratories, Mail Stop: 91R183, Berkeley, CA 94720, USA; Department of Biochemistry, Genetics and Microbiology, Forestry and Agricultural Biotechnology Institute (FABI), University of Pretoria, Private Bag X20, Pretoria 0028, South Africa; Department of Energy Joint Genome Institute, Lawrence Berkeley National Laboratories, Mail Stop: 91R183, Berkeley, CA 94720, USA; Department of Energy Joint Genome Institute, Lawrence Berkeley National Laboratories, Mail Stop: 91R183, Berkeley, CA 94720, USA; Department of Energy Joint Genome Institute, Lawrence Berkeley National Laboratories, Mail Stop: 91R183, Berkeley, CA 94720, USA; Department of Energy Joint Genome Institute, Lawrence Berkeley National Laboratories, Mail Stop: 91R183, Berkeley, CA 94720, USA; Genome Sequencing Center, HudsonAlpha Institute for Biotechnology, 601 Genome Way Northwest, Huntsville, AL 35806, USA; Genome Sequencing Center, HudsonAlpha Institute for Biotechnology, 601 Genome Way Northwest, Huntsville, AL 35806, USA; Arizona Genomics Institute, University of Arizona, 1657 E. Helen St., Tucson, AZ 85721, USA; Research School of Biology and Centre for Biodiversity Analysis, ARC Centre of Excellence in Plant Energy Biology, Australian National University, Canberra, ACT 0200, Australia; Department of Energy Joint Genome Institute, Lawrence Berkeley National Laboratories, Mail Stop: 91R183, Berkeley, CA 94720, USA; Genome Sequencing Center, HudsonAlpha Institute for Biotechnology, 601 Genome Way Northwest, Huntsville, AL 35806, USA; Department of Energy Joint Genome Institute, Lawrence Berkeley National Laboratories, Mail Stop: 91R183, Berkeley, CA 94720, USA; Genome Sequencing Center, HudsonAlpha Institute for Biotechnology, 601 Genome Way Northwest, Huntsville, AL 35806, USA; Department of Ecology and Evolutionary Biology, Institute for Systems Genomics: Computational Biology Core, University of Connecticut, Storrs, CT 06269, USA; Department of Biochemistry, Genetics and Microbiology, Forestry and Agricultural Biotechnology Institute (FABI), University of Pretoria, Private Bag X20, Pretoria 0028, South Africa; Department of Genetics, Stellenbosch University, Private Bag X1, Stellenbosch 7600, South Africa

**Keywords:** *Eucalyptus grandis*, genome improvement, phased assembly, tandem duplications, synteny

## Abstract

*Eucalyptus grandis* is a hardwood tree used worldwide as pure species or hybrid partner to breed fast-growing plantation forestry crops that serve as feedstocks of timber and lignocellulosic biomass for pulp, paper, biomaterials, and biorefinery products. The current v2.0 genome reference for the species served as the first reference for the genus and has helped drive the development of molecular breeding tools for eucalypts. Using PacBio HiFi long reads and Omni-C proximity ligation sequencing, we produced an improved, haplotype-phased assembly (v4.0) for TAG0014, an early-generation selection of *E. grandis.* The 2 haplotypes are 571 Mbp (HAP1) and 552 Mbp (HAP2) in size and consist of 37 and 46 contigs scaffolded onto 11 chromosomes (contig N50 of 28.9 and 16.7 Mbp), respectively. These haplotype assemblies are 70–90 Mbp smaller than the diploid v2.0 assembly but capture all except one of the 22 telomeres, suggesting that substantial redundant sequence was included in the previous assembly. A total of 35,929 (HAP1) and 35,583 (HAP2) gene models were annotated, of which 438 and 472 contain long introns (>10 kbp) in gene models previously (v2.0) identified as multiple smaller genes. These and other improvements have increased gene annotation completeness levels from 93.8 to 99.4% in the v4.0 assembly. We found that 6,493 and 6,346 genes are within tandem duplicate arrays (HAP1 and HAP2, respectively, 18.4 and 17.8% of the total) and >43.8% of the haplotype assemblies consists of repeat elements. Analysis of synteny between the haplotypes and the *E. grandis* v2.0 reference genome revealed extensive regions of collinearity, but also some major rearrangements, and provided a preview of population and pangenome variation in the species.

## Introduction

The eucalypts are a group of woody plants with more than 900 species (Brooker 2000) including a number of fast-growing forestry species that are being domesticated as timber and woody biomass feedstocks for a bio-based economy. Together with natural stands and restoration forestry efforts (Bennett 2016; Jordan *et al*. 2016; Supple *et al*. 2018; Brancalion *et al*. 2020), eucalypt plantations can contribute to global carbon drawdown (Bar-On *et al*. 2018). *Eucalyptus grandis* is the model species for the genus, representing the most widely planted hardwood crop in the world (more than 20 million ha, Iglesias-Trabado and Wilstermann 2008; Myburg *et al*. 2014). *E. grandis* is a highly outbred and heterozygous species (Myburg *et al*. 2014), with a natural habitat spanning much of the east coast of Australia (−16.2494, 145.3213 to −32.9305, 151.7541) and multiple climatic conditions (Mostert-O'Neill *et al*. 2021). Unfortunately, *E. grandis* is highly susceptible to pathogens in subtropical regions (Wingfield *et al*. 2008) and is therefore often used as a hybrid partner, typically with *Eucalyptus urophylla*, for clonal plantations of F1 hybrid varieties in these regions. Due to the commercial importance of *E. grandis* (Harwood 2011) and its position as a genetic model for the genus, numerous genomic (Myburg *et al*. 2014; Bartholome *et al*. 2015; Silva-Junior *et al*. 2015) and transcriptomic (Mizrachi *et al*. 2010; Carocha *et al*. 2015; Mangwanda *et al*. 2015; Oates *et al*. 2015; Soler *et al*. 2015; Vining *et al*. 2015; Hussey *et al*. 2015b; Mizrachi *et al*. 2017) resources have been generated which have greatly aided understanding of the population genetics (Mostert-O'Neill *et al*. 2021) and biology (Mangwanda *et al*. 2015; Oates *et al*. 2015; Soler *et al*. 2015; Hussey *et al*. 2015b; Mizrachi *et al*. 2017; Wierzbicki *et al*. 2019) of *E. grandis*, while promoting the development of molecular breeding tools in the genus (Silva-Junior *et al*. 2015; Silva-Junior and Grattapaglia 2015; Telfer *et al*. 2015; Ballesta *et al*. 2019; Mostert-O'Neill *et al*. 2022; Candotti *et al*. 2023). These resources depend on the current reference genome, which was assembled from whole-genome Sanger and BAC-end sequences of a partially inbred genotype (BRASUZ1) and likely represents a collapsed assembly in the remaining heterozygous regions of the BRASUZ1 genome, rather than a haplotype-phased assembly, which is rapidly becoming the standard in outbred organisms. Despite having proven a very good reference for the applications described above, the v2.0 assembly (Myburg *et al*. 2014) and the improved v2.1 assembly (Bartholome *et al*. 2015) would not have provided robust assessment of genome sequence and structural diversity in the species.

Long-read sequencing technologies promise improved, phased genome assemblies in outbred organisms, along with full-length transcript sequences that result in higher quality annotations. Such phased references allow identification of haplotype-specific variants [including structural variants (SVs)] and the subsequent association of such variants with traits of interest (Tang *et al*. 2021; Cai *et al*. 2022; Shang *et al*. 2022; Vourlaki *et al*. 2022; Scarlett *et al*. 2023; Wang *et al*. 2023; Healey *et al*. 2024). Large SVs contribute to the genetic diversity of the species (pangenome variation) and lead to genome and 3D architectural changes that can alter gene expression regulation (Alonge *et al*. 2020; Kang *et al*. 2023; Ni *et al*. 2023; Ni and Tian 2023; Li *et al*. 2024; Zhang *et al*. 2024). Ultimately, at individual level, such regulatory variation may contribute to phenotypic plasticity, the ability to survive diverse environmental challenges.

Here, we report a haplotype-phased genome reference for *E. grandis*, which is highly contiguous and nearly complete. This improved reference provides an important resource for studies focusing on genome sequence and structural evolution in the species compared to earlier studies that relied on sparse SNP markers alone (Mostert-O'Neill *et al*. 2021, 2022). In addition, as an early-generation commercial clone, the TAG0014 reference will be useful for studies of the genetic consequences of early domestication and breeding. Together with other recent phased genome assemblies in the genus (Ferguson *et al*. 2024a, 2024b, 2024c), the phased *E. grandis* (TAG0014, v4.0) assembly and improved gene annotation will also improve understanding of gene function and evolution and support tree biotechnology efforts aimed at improving tree growth, development, and resilience to climate change and associated biotic challenges.

## Methods and materials

### Genome sequencing and haplotype-phased assembly

We sequenced an early selection (first generation from wild, unimproved material) of *E. grandis* which has been clonally propagated since the 1990s but is no longer used as a commercial clone. The availability of ample clonally replicated trees (Mondi South Africa) allowed us to perform whole-tree collection of developing tissues for genome and transcriptome analysis. As a clone that was derived from early breeding trials involving seed from unimproved material from several provenances, TAG0014 is possibly an inter-provenance cross of *E. grandis* with higher heterozygosity than individuals from native provenance stands. It was selected to be the new reference due to its early-generation status and availability as a clonal genotype for molecular studies and because of the numerous data sets already generated for this clone (Vining *et al*. 2015; Hussey *et al*. 2015a, 2017).

The TAG0014 genome was sequenced with a range of sequencing technologies including PacBio HiFi (Pacific Biosciences Inc., CA, USA), chromosome conformation capture with Omni-C (Dovetail Genomics, CA, USA), and Illumina short-read sequencing data. PacBio Iso-Seq and Illumina RNA-seq data were generated for genome annotation. High molecular weight DNA was isolated from frozen leaf tissue at the Arizona Genomics Institute (AGI, Tucson, Arizona, USA). PacBio and Illumina sequence reads were generated at the HudsonAlpha Institute for Biotechnology (HA) in Huntsville, Alabama, and at the Department of Energy Joint Genome Institute (DOE-JGI) in Berkeley, California. An Illumina library (400 bp insert) was generated and sequenced on the NovoSeq 6000 platform to obtain 2 × 150 bp paired-end reads to a coverage of 64.4 × along with a 2 × 150 nt Dovetail Omni-C library (Supplementary Fig. 1). PacBio sequence reads were generated using the Sequel IIe platform at HA.

PacBio HiFi reads (41.92 Gbp, 65.50 × coverage, and read N50 of 17.74 kbp) were assembled into initial haplotype-phased contigs using HiFiAsm + HiC (Cheng *et al*. 2021). The assemblies were polished with RACON (Vaser *et al*. 2017), and no misjoins were identified in the polished assemblies. This produced highly contiguous initial assemblies for both haplotypes. The assemblies were further scaffolded, ordered, and oriented using Omni-C proximity ligation contacts generated with the JUICER pipeline (Durand *et al*. 2016) and the *E. grandis* v2.0 genome. Contigs terminating in significant telomeric sequences were properly oriented in the assembly. For HAP1, a total of 13 joins resulted in the final assembly of 11 chromosomes (containing 99.991% of the assembled sequence) and 1 small unplaced scaffold of 50 kbp. Adjacent alternative haplotypes were identified on the joined contig set. Seven adjacent alternative haplotype (althap) regions were collapsed in the assembly using the longest common substring between the 2 haplotypes. Similarly, for HAP2, 40 joins were applied to obtain a final assembly of 11 chromosomes which contains 100% of the assembled sequence. A total of 16 adjacent althap regions were collapsed in the HAP2 assembly. The resulting assemblies were screened for contaminants using NCBI's FCS-GX pipeline (Astashyn *et al*. 2024). The mitochondrial and chloroplast genomes were assembled using OatK (https://github.com/c-zhou/oatk). Additional unanchored scaffolds were classified as redundant if >95% of the assembled contig sequence was masked with 24mers that occurred 3 or more times (63/100 HAP1/HAP2 scaffolds contained ≥95% of 24mers occurring >2 × in all scaffolds, 8.9/13.9 Mbp, respectively), repetitive if unanchored contigs have >95% of its sequence masked by 24mers that occurred 4 or more times (39/21 ≤250 kbp scaffolds contained ≥95% of 24mers occurring >4× in ≥5 Mbp scaffolds, 3.9/1.5 Mbp, respectively), or organellar (assembled with OatK before genome assembly, 456.9 kbp mitochondrial genome, 160.2 kbp chloroplast).

HifiAsm may occasionally misphase small regions within the chromosomes (Healey *et al*. 2024). Misphased regions in the TAG0014 haplotypes (v3.0) were identified through alignment of Omni-C data to the combined HAP1/HAP2 chromosomes and manual identification of the misphased regions in the contact map (Supplementary Table 4). The corrected haplotype-specific phased assemblies were the final assemblies selected as haplogenome references (v4.0). The genome assemblies were screened for PacBio linkers, and 14/13 instances of linkers were found in HAP1/HAP2 (Supplementary Table 4). When comparing haplotypes, we noted regions on Chr01 HAP2 that were absent in HAP1. Closer inspection revealed that the HAP2 region had 2-fold higher depth than the surrounding regions suggesting that it was likely a collapsed homozygous copy. In the v4.0 TAG0014 release, the entire homozygous region identified by a doubling of CCS depth was duplicated in HAP1 (both haplotypes were updated to include a single homozygous copy of the previously collapsed copy represented in HAP2 on chromosome 1 position 1–1,162,920).

Homozygous SNPs and INDELs were corrected in the HAP1 and HAP2 releases using ∼60.4 × Illumina reads (2 × 150, 400 bp insert) by aligning the reads to each haplotype using bwa mem (Li and Durbin 2010) and identifying homozygous (haploid) SNPs and INDELs with the GATK's UnifiedGenotyper tool (McKenna *et al*. 2010). Homozygous SNPs/INDELs were then corrected using an in-house developed pipeline, and the Illumina reads were re-aligned to the corrected sequence to verify the corrections. A total of 68/118 (HAP1/HAP2) SNPs and 3,753/4,048 INDELs were corrected for HAP1/HAP2, respectively. Heterozygous SNPs and INDELs were corrected using 47.75 × CCS data, fixing 90/44 SNPs and 654/627 INDELs for HAP1/HAP2, respectively.

Genome assembly statistics were generated with QUAST v5.2.0 (Gurevich *et al*. 2013; Mikheenko *et al*. 2018), and completeness was assessed with BUSCO v5.4.5 (Simao *et al*. 2015; Seppey *et al*. 2019; Manni *et al*. 2021) using the embryophyta_odb10 database. Genome contiguity of the TAG0014 phased (v4.0) and v2.0 diploid genome reference was visualized with GENESPACE (Lovell *et al*. 2022). In addition, *k*-mer-based quality of the assemblies was assessed using Meryl v1.3 and Merqury v1.3 (Rhie *et al*. 2020) using default parameters.

Completeness of the euchromatic portion of the genome assembly was assessed by aligning 34,121 annotated primary transcripts from the *E. grandis* v2.0 annotated primary transcripts to the v4.0 HAP1 and HAP2 release. The completeness analysis aims to obtain a measure of completeness of the assembly, rather than a comprehensive examination of the gene space. We retained genes that aligned at greater than 90% identity and 85% coverage. We found that 32,663/32,577 (95.73%/95.47%) of the previously annotated genes align to the HAP1/HAP2 v4.0 release. Of the remaining annotated genes, 1,068/1,171 (3.13%/3.43%) aligned at <50% coverage and 390/373 (1.14%/1.09%) of genes were not found in the HAP1/HAP2 v4.0 release.

### Genome annotation

Total RNA was extracted from 15 tissues representing different developmental stages and positions on the TAG0014 tree (Supplementary Fig. 1) using the Plant/Fungi total RNA purification kit (Norgen Biotek Corp.) per the manufacturer's protocol. RNA purity was assessed using spectroscopy on a NanoDrop ND1000 (Nano-Drop Technologies), and integrity was verified with an Agilent 2100 Bioanalyzer (Agilent Technologies, Santa Clara, CA, USA). RNA-seq data were generated by the JGI (Berkeley, CA) for the 15 tissue libraries prepared using Illumina's TruSeq Stranded mRNA HT sample prep kit using poly-A selection of the mRNAs. The libraries were sequenced on the NovaSeq S4 platform to produce 2 × 150 bp RNA-seq reads. Transcripts were assembled from ∼367M 2 × 150 bp stranded PE reads with PERTRAN (Shu *et al*. 2013), which conducts genome-guided transcriptome short-read assembly via GSNAP (Wu and Nacu 2010) and builds splice alignment graphs after alignment validation, realignment, and correction.

PacBio Iso-Seq full-length RNA transcript sequences were generated using the Sequel IIe platform for 3 tissue pools (developing cambium/woody, green/leaf and flower) generated using pools of the RNA extracted above. A genome-guided correction pipeline was used to correct and collapse 25.9 M PacBio Iso-Seq CCS reads. The pipeline aligns CCS reads to the genome with GMAP (Wu and Nacu 2010), corrects small INDELs in splice junctions, and clusters alignments when all introns are the same or ≥95% overlap for single-exon alignments. This resulted in ∼915 K/908 K putative full-length transcripts for HAP1 and HAP2, respectively. Transcript assembly sets were combined into a final set of 364,350 HAP1 transcripts and 359,285 HAP2 transcripts with PASA (Haas *et al*. 2003).

A *de novo* repeat library was created for the TAG0014 HAP1 v3.0 genome draft using RepeatModeler2 (Flynn *et al*. 2020). To identify repeats with significant hits to protein-coding domains, the repeat library was functionally analyzed with InterProScan (Jones *et al*. 2014) based on the Pfam (Mistry *et al*. 2021) and PANTHER (Mi *et al*. 2019) databases. Repeats with significant protein-coding domain hits were removed from the repeat library, and the genome was soft-masked using RepeatMasker (Smit *et al*. 2013) with the resulting species-specific repeat library.

To determine putative gene loci, transcript assembly alignments and/or EXONERATE (Slater and Birney 2005) alignments of proteins from *Corymbia citriodora*, *Arabidopsis thaliana*, *Glycine max*, *Fragaria vesca*, *Vitis vinifera*, *Liriodendron tulipifera*, *Punica granatum*, *Rhodamnia argentea*, *Syzygium oleosum*, *Brassica rapa*, *Citrus clementina*, *Gossypium raimondii*, *Populus trichocarpa*, *Oryza sativa*, *Beta vulgaris*, and Swiss-Prot release 2022_04 of eukaryote proteomes were generated using repeat-soft-masked *E. grandis* var. TAG0014 HAP1 and HAP2 v4.0 genomes, with up to 2 kbp bidirectional extension unless extending into another locus on the same strand. Gene models in each locus were predicted by homology-based predictors, FGENESH+ (Salamov and Solovyev 2000), FGENESH_EST (similar to FGENESH+, but using PASA-assembled transcripts to compute splice site and intron input instead of protein/translated ORF), EXONERATE, PASA assembly ORFs (in-house homology constrained ORF finder), and AUGUSTUS (Stanke *et al*. 2006) trained on the high confidence PASA assembly ORFs and with intron hints from short-read alignments. The best-scored predictions for each locus were selected using multiple positive factors including transcriptome and protein support and 1 negative factor: overlap with repeats. The selected gene predictions were improved by adding UTRs, splicing correction, and adding alternative transcripts with PASA.

To obtain a Cscore (the protein BLASTP score ratio to the mutual best hit BLASTP score) and protein coverage (the percentage of protein aligned to the best of homologs), PASA-improved gene model proteins were subject to protein homology analysis to the above-mentioned proteomes. PASA-improved transcripts were selected based on Cscore, protein coverage, transcriptome coverage, and their CDS overlap with repeats. The transcripts were selected if their Cscore and protein coverage were ≥0.5 or if covered by transcripts assembled by PASA. For gene models whose CDS overlapped with repeats predicted by RepeatMasker by more than 20%, the Cscore had to be at least 0.9 and homology coverage at least 70% to be selected. The selected gene models were subject to Pfam analysis, and gene models without strong transcriptome and homology support and whose proteins overlapped more than 30% with Pfam transposable element (TE) domains were removed. Incomplete gene models, low homology supported without full transcriptome-supported gene models, short single exon (<300 bp CDS) without protein domains supported by less than 15 RNA-seq reads, and genes with high copy numbers (more than 16 copies) without strong homology support were manually filtered out.

Detailed gene metrics were obtained for the TAG0014 haplotypes and for the v2.0 genome with gFACs v1.1.2 (Caballero and Wegrzyn 2019). Completeness of transcript and protein models was determined using BUSCO v5.4.5 and the embryophyta_odb10 database.

### Improvement of reference genome

The improvement of the v4.0 TAG0014 phased reference over that of the v2.0 draft was evaluated based on the assembly and annotation completeness, number of gene models, assembly contiguity, genome feature presence, and whether the assembly was phased and by looking for assembly gaps that have been closed. Telomere sequences were identified for all genomes using the (CCCGAAA)n and (CCCTAAA)n repeats with GENESPACE. Overall conservation of genome structure was evaluated through genome-wide and gene-based synteny analyses.

Genome-wide synteny was assessed with SyRI (Goel *et al*. 2019). Pairwise combinations of reference genome assemblies were aligned using nucmer from the MUMmer4 toolbox (Marcais *et al*. 2018). Resulting alignments were filtered with nucmer (“—maxmatch –c 100 -b 500 -l 50”). The alignments were further filtered for alignment length (>100) and identity (>90), then used to identify local variants (LVs) and structural rearrangements with SyRI v1.6.3, and visualized with plotsr v1.1.1 (Goel and Schneeberger 2022).

The overall conservation of synteny of orthologous and homeologous gene regions was assessed using GENESPACE (Lovell *et al*. 2022). GENESPACE is based on protein similarity scores to construct synteny blocks with MCScanX (Wang *et al*. 2012) and Orthofinder v2.5.5 (Emms and Kelly 2015; 2019). Syntenic blocks were used to identify pairwise peptide differences among the *E. grandis* genomes.

One of the main features of the v2.0 reference genome was the high percentage of tandem duplicate arrays (12,570 genes in 3,185 clusters, Myburg *et al*. 2014). Since technological and methodological advances have been made since the original publication, we reevaluated tandem duplicate gene content across all of the *E. grandis* reference genomes. Using the “combed.txt” output generated from GENESPACE, all orthogroups with more than 2 genes were considered as a tandem array and the number of genes and number of arrays were extracted using custom R scripts. The number of genes per array was also compared to find arrays where there are expansion/contractions relative to the current v2.0 genome reference.

### Population diversity analyses

The genetic relatedness of the TAG0014 genome to natural populations (provenances) of *E. grandis* was assessed using a 72 K SNP Axiom 384-format array for *Eucalyptus* and *Corymbia* (Affymetrix Inc., Santa Clara, California, USA). Allelic intensity data were first assessed using Axiom Analysis Suite v5.2.0.65 to recluster genotypic classes as described by (Silva-Junior *et al*. 2015). SNP data for natural population references were obtained from Mostert-O'Neill *et al*. (2021), and the SNP calls were converted to Axiom SNPs using a custom R script in RStudio v2023.12.1. The converted calls were imported into SNP & Variation Suite v8.9.1 (SVS8; Golden Helix, Inc., Bozeman, MT). High confidence, informative SNPs were retained by filtering for minor allele frequency > 0.02, genotyped in at least 80% of samples and Hardy Weinberg Equilibrium *P* > 1e−5. Starting with 18,328 SNP markers that mapped to the v2.0 reference genome, 17,036 SNPs remained after filtering. To determine the genetic relatedness of the TAG0014 genome with trees representing the natural range of *E. grandis*, SNP data from Mostert-O'Neill *et al*. (2021) and the TAG0014 genome were used to perform a principal component analysis (PCA) (Price *et al*. 2006) in SVS8.

## Results

### Genome assembly and annotation

**Fig. 1.**
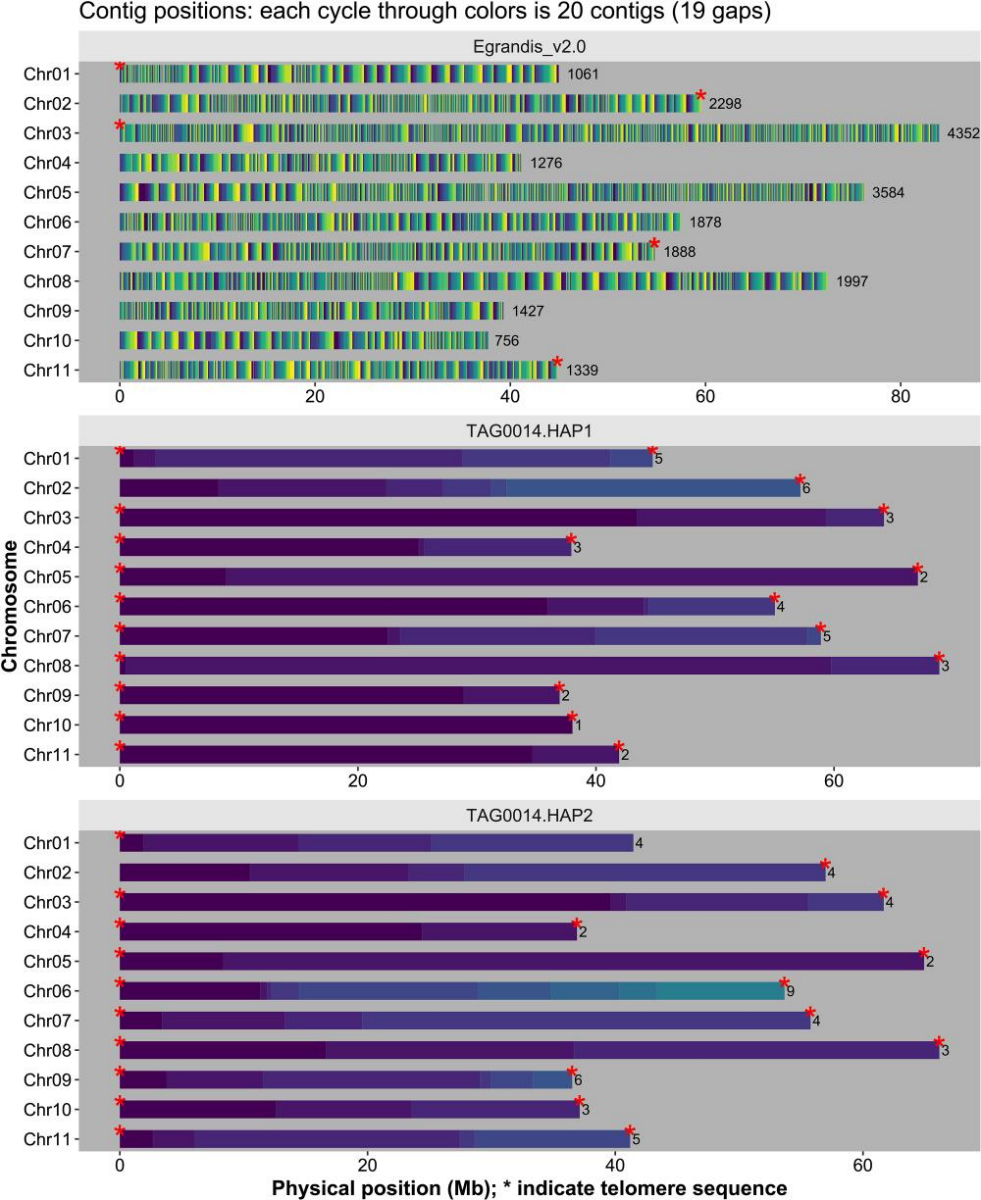
Improvement in contiguity of the *E. grandis* reference genome. Contig position of the v4.0 reference genome assemblies was generated using GENESPACE (Lovell *et al*. 2022). The contigs in the previous *E. grandis* v2.0 reference assembly (top panel), TAG0014 HAP1 assembly (middle panel), and TAG0014 HAP2 assembly (bottom panel) are shown as continuous blocks of a single color. A blue-yellow palette of 20 colors was selected for all assemblies; i.e. each cycle through colors represents 20 contigs. Telomere sequences are shown by a red asterisk, and the numbers of contigs that make up individual chromosome scaffolds are shown on the right. Chromosome position and size are indicated on the *x* axis and chromosome number on the *y* axis.

The *E. grandis* v2.0 reference had some limitations that prohibited its use to study larger SVs that contribute to pangenome variation in the species. First, due to the sequencing technology used and genome assembly capabilities available at the time, repeat regions were difficult to resolve. As a result, the v2.0 genome reference was a fragmented assembly consisting of 21,856 contigs in 4,943 scaffolds (Fig. 1 and Table 1). Of the 640.4 Mbp v2.0 scaffolded reference, 7.4% (47.4 Mbp) is contained in gaps which may be the result of an overinflated genome size estimate (i.e. a forced genome size fit based on C-size estimates) and, to some extent, the retention of heterozygous genomic regions in the assembly. Recent studies suggest a smaller genome size when haplotype-phased genome assemblies are generated (Lotter *et al*. 2022; Shen *et al*. 2023).

**Table 1.** Genome assembly and annotation statistics for the *E. grandis* v2.0 reference and the new phased TAG0014 (v4.0) HAP1 and HAP2 assemblies.

	*E. grandis* v2.0	TAG0014 HAP1	TAG0014 HAP2
Assembly size (Mbp)	691.3	570.8	552.4
Number of contigs	32,835	37	46
Contig N50 (Mbp)	0.0672	28.9	16.7
Number of scaffolds	4,943	12	11
Scaffold N50 (Mbp)	57.5	57.2	55.8
Assembly BUSCO (%)	97.9	98.5	98.3
QV		55.1	55.7
Number of telomeres	5	21	20
Genome in scaffolds > 50 kbp (%)	94.2	100	100
Number of genes (primary transcripts)	36,349	35,929	35,583
Number of mono-exonic genes	7,611	6,182	6,165
Number of multi-exonic genes	28,738	29,747	29,418
Repeat content (%)	44.5	44.99	43.78
Number of genes with long introns (>10 kbp)		438	472
Alternative transcripts	9,931	28,647	28,368
Annotation BUSCO (%)	93.8	99.4	99.5

TAG0014 is highly heterozygous, with ∼5.2 million heterozygous SNPs and ∼534,000 heterozygous small INDELS across the 2 TAG0014 haplotypes (Supplementary Table 3), further motivating the establishment of a new reference for the species. We sequenced and assembled 570.8 and 552.4 Mbp for HAP1 and HAP2 of TAG0014, respectively. Both assemblies contained 11 chromosomes, with a contig N50 of 28.9/16.7 Mbp, a scaffold N50 of 57.2/55.8 Mbp, and QV of 55.1/55.7, respectively (Fig. 1 and Table 1; Supplementary Table 1). The haplotypes included 21/20 of the 22 telomeres supporting the improved completeness of the assembly (Fig. 1 and Table 1). Genome assembly completeness was >98.3%, with only 3.2%/2.5% duplicated complete BUSCOs, reflecting the haploid nature of the assemblies (Supplementary Fig. 2 and Table 2).

The HAP1/HAP2 genomes consisted of 44.99%/43.78% TEs (Table 1; Supplementary Table 5). Most repeats were unknown elements (27.51%/26.34% of the genome), followed by *Copia* LTRs (8.96%/9.18%) and *Gypsy* LTRs (3.58%/3.45%) (Fig. 3 and Table 1; Supplementary Table 5). LTR retrotransposons had an average length of 1,327/1,324 bp for HAP1/HAP2.

A combination of *ab initio* prediction, transcript evidence from PacBio Iso-Seq data of 3 tissue pools, and short-read RNA-seq data from 15 tissues was used to predict protein-coding regions. We annotated 35,929 and 35,583 protein-coding genes, with 28,647 and 28,368 alternative transcripts in HAP1 and HAP2, respectively (Fig. 3 and Table 1; Supplementary Table 6). We found 6,182 and 6,165 mono-exonic genes for HAP1 and HAP2, respectively (Supplementary Table 7). Similarly, there were 29,747 and 29,418 multi-exonic genes for HAP1 and HAP2 (Supplementary Table 7). The average number of exons per gene was 5.1 for HAP1 and 5.0 for HAP2 (Supplementary Table 6). More than 99.4% of the 1,614 embryophyta_odb10 BUSCO genes were annotated (Supplementary Table 2). The average genome-wide gene density was 1 gene every 15.89 kbp in HAP1 and 1 gene every 16.04 kbp in HAP2 with an average gene length of 4,020 bp (HAP1) and 3,994 bp (HAP2) and a maximum intron length of 87,554/39,127 (HAP1/HAP2) bp (Supplementary Table 7). A total of 438 and 472 genes contain introns larger than 10 kbp (Table 1). We found that a total of 1,154/1,137 genes from the v2.0 reference were merged into 544/536 genes in TAG0014 HAP1/HAP2, of which 200/189 genes were merged into 82/75 genes with long introns (>10 kbp).

### An improved reference genome for *E. grandis*

The 2 haplotype assemblies were approximately 70–90 Mbp smaller than the v2.0 reference. The assemblies also contained fewer gaps (0.1%), compared to the 7.4% of gaps observed in the v2.0 reference. Overall, the 2 assemblies were over 700-fold more contiguous than the v2.0 reference in terms of number of contigs (i.e. 700-fold fewer contigs; Fig. 1). This is due to a combination of HiFi long-read technology, much higher sequencing depth (65.5×), and the use of Omni-C contact maps which are more informative for fine-scale genome scaffolding than the genetic linkage maps used for the v2.0 assembly. Finally, the assembly includes 21 and 20 of the 22 telomeres in each haplotype, which is 4-fold more than the v2.0 reference (Fig. 1 and Table 1).

The fact that we used the same sequencing and assembly methodologies for the 2 haplotypes with similar accuracy, completeness, and contiguity facilitated direct comparison of the 2 haplotypes to assess differences in genome features that better reflect haplotype divergence in *E. grandis* than previous studies. We acknowledge that comparison of genome-wide synteny based on pairwise assembly alignments with the current v2.0 reference genome most likely reflect assembly differences rather than true genetic differences and caution against evolutionary interpretations from these results. Therefore, when making biological interpretations and conclusions regarding synteny, we only considered comparisons between the TAG0014 HAP1 and HAP2 assemblies.

**Fig. 2.**
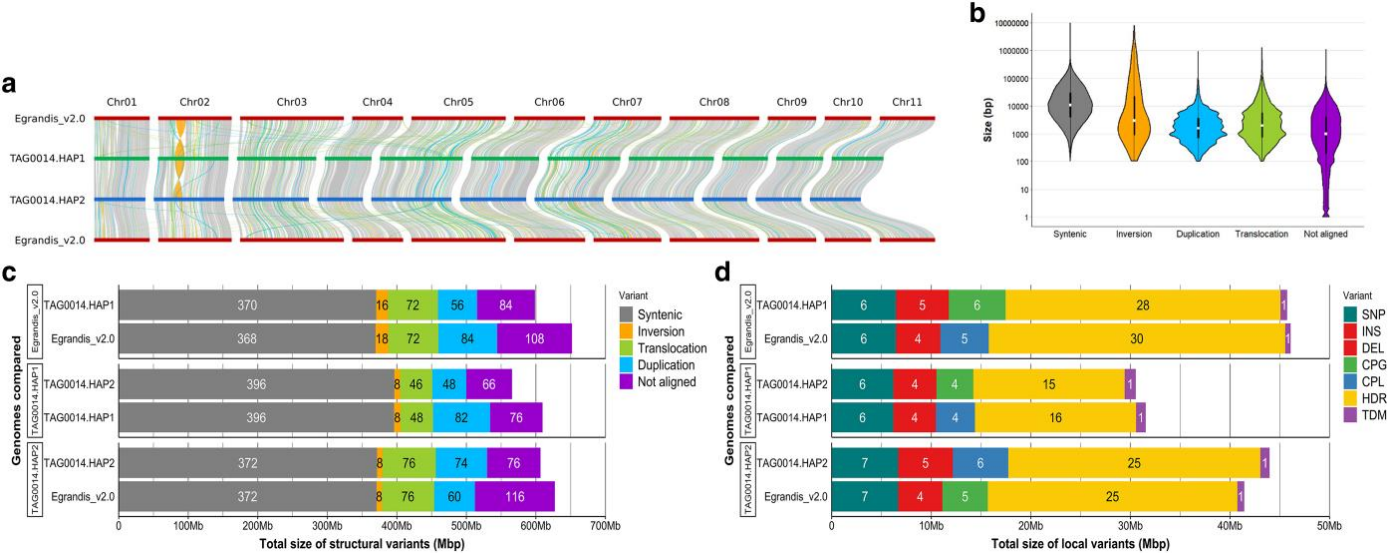
Distribution and size of structural rearrangements and LVs. a) Position of SVs across and between chromosomes of the v2.0 reference assembly, TAG0014 HAP1, and TAG0014 HAP2 as calculated with SyRI (Goel *et al*. 2019) and visualized with plotsr (Goel and Schneeberger 2022). Chromosome numbers are indicated at the top. SV colors are indicated in the same color scheme as shown in b) and c). b) Size distribution of syntenic regions, SVs, and regions that do not align. Variant type is shown on the *x* axis and size in base pairs on the *y* axis (on a logarithmic scale). c) Total size contribution of syntenic regions, SVs, and nonaligned regions for each pairwise assembly comparison. The reference genome for each comparison is provided as facet labels on the *y* axis, along with the specific genome compared. Total size of regions comprising each the variant type is indicated on the *x* axis in megabase pairs. d) Size contribution of LVs for each pairwise assembly comparison. The reference genome for each comparison is provided as facet labels on the *y* axis, along with the specific genome compared. The total size of the variant type is indicated on the *x* axis in megabase pairs. Variants compared are SNPs, insertions (INS), deletions (DEL), copygains (CPG), copylosses (CPL), highly diverged regions (HDR), and tandem repeats (TDM).

We performed pairwise genome synteny comparisons as defined by SyRI (a sequence collinearity-based metric) (Goel *et al*. 2019) between the *E. grandis* v2.0 and TAG0014 HAP1, TAG0014 HAP1 and TAG0014 HAP2, and TAG0014 HAP2 and *E. grandis* v2.0 genome assemblies. We found that 370.2/370.7 Mbp (approximately 53.6%) of the HAP1/HAP2 assemblies were syntenic to the v2.0 genome assembly. In comparison, the HAP1 and HAP2 assemblies were more syntenic to each other with 396.0/396.3 Mbp (69.4%/71.7%) being syntenic (Fig. 2; Supplementary Table 8). This is expected for genome assemblies generated using the same sequencing and assembly methods and illustrates why caution should be applied when comparing genomes sequenced and assembled with different technologies. Overall, the *E. grandis* genomes were largely collinear with 1,448–1,474 translocations (total 51.0 and 55.1 Mbp) and 38–39 inversions larger than 10 kbp (total 18.2 and 7.6 Mbp; Fig. 2). In comparison, the TAG0014 genomes were more collinear with 1,018 translocations (29.3 Mbp) and 22 inversions (9.25 Mbp) larger than 10 kbp between the 2 genomes. We observed more regions that do not align between the v2.0 reference and the HAP1 (107.7 Mbp, 15.6%) and HAP2 (114.9 Mbp, 16.6%) assemblies than between HAP1 and HAP2 (65.8–75.5 Mbp, 11.9–13.2%; Fig. 2; Supplementary Table 8). In addition, there were fewer translocations and duplications between HAP1 and HAP2 than between either of them and the v2.0 reference assembly (Supplementary Table 8). This may reflect the difficulty of assembling and collapsing such regions into a single haplotype representation of the diploid genome of outbred organisms such as *E. grandis*. The size of SVs ranged from 100 bp to 7.96 Mbp, with the largest event detected being an inversion between HAP1 and HAP2 of TAG0014 (Fig. 2).

We observed fewer LVs (smaller than 50 bp within larger alignment blocks, including insertions, deletions, SNPs, copygains, copylosses, tandem repeats, and highly diverged regions) between the HAP1 and HAP2 assemblies than between the TAG0014 assemblies and the v2.0 reference genome. For LVs, there are almost twice as many highly diverged regions (single base pairs missing in both assemblies within an alignment block) between the v2.0 reference and TAG0014 than between HAP1 and HAP2 of TAG0014. We also noted more SNPs (6.5 million vs 6.2 million), insertions (4.9 million vs 4.3 million), deletions (4.9 million vs 4.3 million), copygains (1,516 vs 1,274), copylosses (1,533 vs 1,219), and tandem repeats (320 vs 286) on average between the v2.0 reference and the TAG0014 haplotypes than between the TAG0014 haplotypes (Fig. 2; Supplementary Table 9). The total size of LVs was larger when comparing TAG0014 haplotypes to the v2.0 reference (45.9 Mbp for HAP1 and 42.7 Mbp for HAP2) than between the TAG0014 haplotypes (31.0 Mbp; Fig. 2; Supplementary Table 9).

**Fig. 3.**
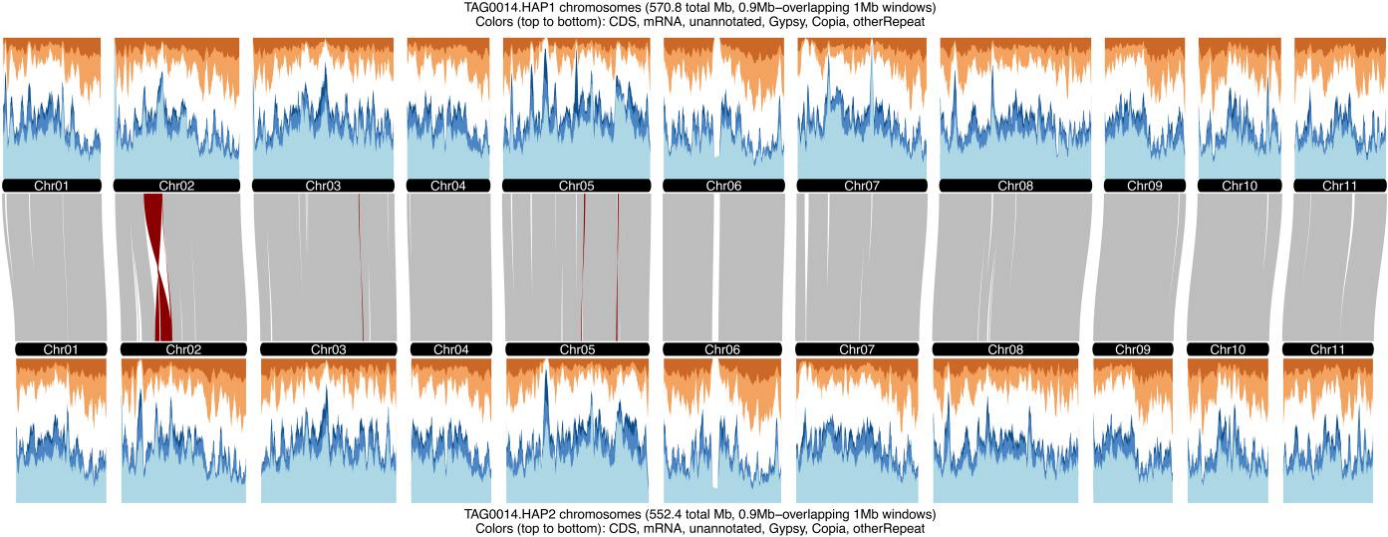
Structure and genome features of the 2 haplotypes of the phased TAG0014 (v4.0) genome assembly. The genome architecture between the TAG0014 HAP1 and HAP2 assemblies generated with GENESPACE. Repeat and gene density were hierarchically classified as coding sequences (CDS, dark orange), mRNA (light orange), Gypsy repeats (dark blue), Copia repeats (mid blue), other repeats (light blue), and other (white). Sliding windows of 1 Mbp are shown with 0.9 Mbp steps plotted along the horizontal axis.

The TAG0014 haplotypes had similar gene content with 128.48 Mbp (HAP1) and 126.93 Mbp (HAP2) while the gene content of the v2.0 reference was lower at 111.95 Mbp. This may also explain the lower BUSCO completion score of the v2.0 genome annotation. The repeat content of the v2.0 reference (44.5%) was similar to that of the TAG0014 assemblies (44.9% HAP1/43.78% HAP2; Fig. 3 and Table 1; Supplementary Table 5).

Using a gene-based synteny approach, we detected high levels of collinearity between the v2.0 reference genome and the TAG0014 haplotypes and between the TAG0014 haplotypes (Fig. 3). We found 101,490 genes in 21,149 phylogenetically hierarchical orthogroups (HOGs produced by Orthofinder), of which 16,144 were single-copy HOGs shared by all 3 assemblies, while 2,602 genes in 669 HOGs were assembly-specific and 6,371 genes were not assigned to a HOG.

One of the main features of the *E. grandis* v2.0 reference genome was the high number of genes in tandem duplicate arrays. Tandem duplicate arrays are still difficult to identify, but since the release of the v2.0 reference genome, new methods have been developed to define such arrays more accurately. Our analyses using GENESPACE revealed 2,235, 2,232, and 2,106 arrays for HAP1, HAP2, and v2.0 that contained 6,493, 6,346, and 5,488 genes, respectively (Supplementary Table 10). This was fewer than the 12,570 (34%) genes described for the v2.0 reference but still made up 18.40, 17.83, and 15.10% of the respective total number of genes (Supplementary Table 10). Further analyses revealed that the v2.0 reference genome has larger arrays (with more genes in the array) than TAG0014 HAP1/HAP2 for 1,799/1,804 OGs and smaller arrays for 2,047/2,053 OGs, with 1,726 arrays larger than both haplotype-phased assemblies and 1,764 smaller than both.

### TAG0014 diversity representation

**Fig. 4.**
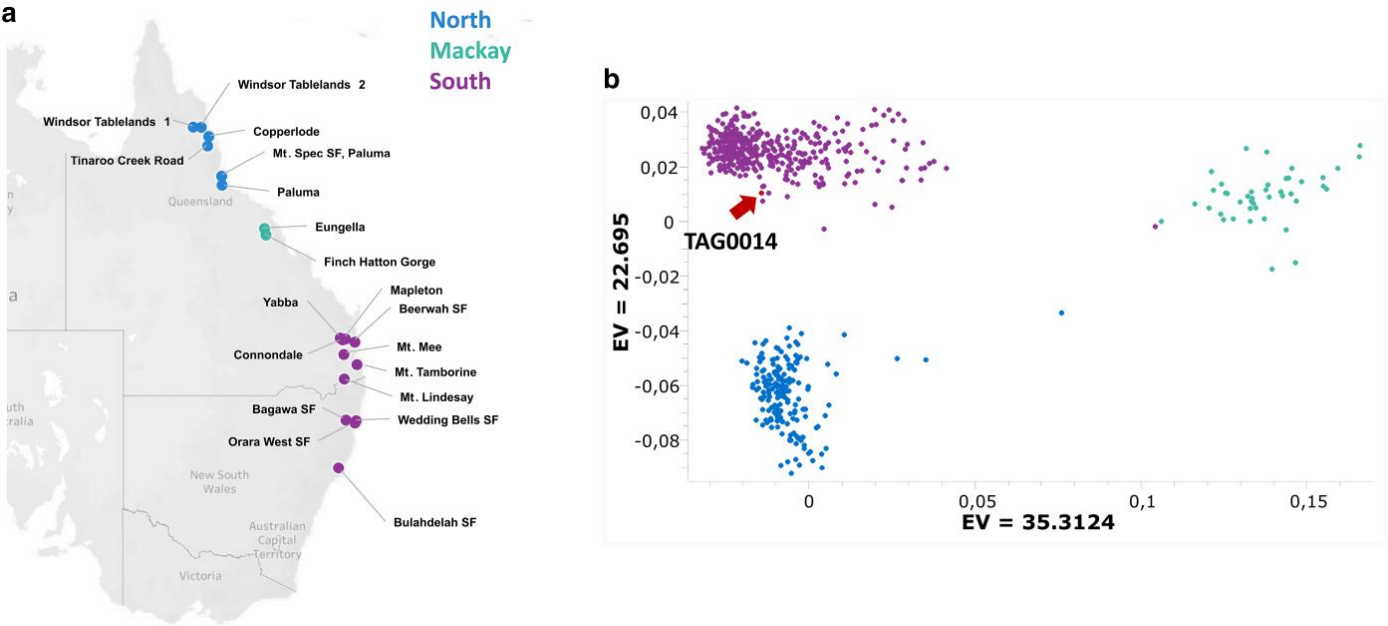
Genetic relatedness of the reference individual TAG0014 compared to the natural distribution and population diversity of *E. grandis* [modified from Mostert-O'Neill *et al*. (2021)]. a) Broad geographic regions where *E. grandis* is found. Provenances are colored and grouped by broad geographic regions, North (blue), Mackay (green), and South (purple). b) Population structure is depicted based on PCA with TAG0014 included as shown by the red arrow based on 17,036 SNPs.

By comparing its SNP profile to that of 33 previously genotyped provenances (Mostert-O'Neill *et al*. 2021), we found that TAG0014 clusters with the wild genotypes from the Southern part of the natural range (Fig. 4). As a first-generation selection from wild, unimproved material, we expected TAG0014 to cluster closely to a specific geographic region (Mostert-O'Neill *et al*. 2022).

## Discussion

The current collapsed diploid genome reference for *E. grandis* (v1.1: BRASUZ1; Myburg *et al*. 2014, later improved by Bartholome *et al*. 2015; v2.0) has limitations for studying large SVs that contribute to pangenome diversity in the species. Due to the technologies and methodologies used for genome sequencing and assembly, the v2.0 assembly likely contains many instances of co-assembly of partially overlapping alternative haplotypes collapsed into a pseudo-haploid reference sequence with many cases of haplotype switching in the heterozygous regions of the genome. Although TAG0014 clusters within the diversity of the natural range of *E. grandis*, the TAG0014 reference is not representative of all natural diversity in *E. grandis* (Fig. 4) pointing to the need for additional provenance and pangenome references to fully understand the natural genome diversity of *E. grandis.* Using a combination of PacBio HiFi, Illumina, Omni-C, RNA-seq, and PacBio Iso-Seq sequencing data, we produced an improved, haplotype-phased reference genome assembly and annotation for *E. grandis.* The phased v4.0 reference will enable studies aimed at understanding the effects that sequence and SVs have on gene regulation and local adaptation and provide access to a new source of genetic variation for molecular breeding.

The v4.0 haplotype-phased assemblies are 70–90 Mbp smaller than the v2.0 reference, likely reflecting that the collapsed diploid assembly is inflated relative to the 2 haplotype-phased assemblies in the v4.0 reference. Other recent phased assemblies for *E. grandis* show a similar trend (Lotter *et al*. 2022; Shen *et al*. 2023). The TAG0014-phased assemblies are more contiguous and complete than the v2.0 reference, with almost no gaps and almost all telomeres assembled. With the aid of proximity ligation data (Omni-C), all contigs could be placed onto the 11 chromosomes of each haplotype assembly.

We find that the TAG0014 haplotypes have similar number of genes to that of the v2.0 reference (35,929/35,583 HAP1/HAP2 compared to 36,349 in v2.0) and have discovered 438 and 472 genes that have introns longer than 10 kbp. These genes merge 200/189 of the original v2.0 gene models and were only discoverable due to the use of PacBio Iso-Seq long-read RNA sequencing data. The v4.0 gene models are also more complete than the current v2.0 annotation (>99.5% vs 93.8%) and with fewer duplicate genes (3.5%/2.8% HAP1/HAP2 vs 5.0% for v2.0; Supplementary Table 2). The repeat content of the TAG0014 assemblies is similar to those of the v2.0 assembly (44.99%/43.78% HAP1/HAP2 vs 44.5% for v2.0; Table 1; Supplementary Table 5). This suggests that the change in assembly size is not due to a general change in the repeat content and that the repeat content is well captured. This also suggests that the smaller TAG0014 genome size is due to more accurate assembly of complex, heterozygous regions vs the need to collapse such regions in the v2.0 pseudo-haploid assembly.

One of the outstanding genomic features of the v2.0 reference was the large proportion of tandem duplicate genes, the largest observed for any sequenced plant genome at the time (Myburg *et al*. 2014). We have used improved methodologies to assess this feature (GENESPACE) and found that there are fewer genes in tandem arrays than previously stated [5,488 in v2.0 compared to 12,570 claimed by Myburg *et al*. (2014); Supplementary Table 10]. However, even though there are fewer tandem duplicate arrays than claimed previously, our GENESPACE analysis indicated that the haplotype-phased TAG0014 assemblies have more genes in tandem arrays than the v2.0 reference (approximately 1,000 more genes in tandem arrays, with 6,493 and 6,346 genes contained in 2,235 and 2,232 arrays for HAP1 and HAP2, compared to 5,488 genes in 2,106 arrays for the v2.0 reference) which comprise 18.40 and 17.83% of the genes, respectively.

## Supplementary Material

jkaf112_Supplementary_Data

## Data Availability

The *E. grandis* v2.0 reference is available at https://phytozome-next.jgi.doe.gov/. The TAG0014 HAP1 and HAP2 genomes are available on Phytozome (https://phytozome-next.jgi.doe.gov/) and can be downloaded here: https://data.jgi.doe.gov/refine-download/phytozome?q=Eucalyptus+grandis+var.+TAG0014. The raw data is available on NCBI under BioProject PRJNA1217046. Scripts for genome comparisons and summary statistics and to generate images are available on GitLab: https://gitlab.com/Anneri/tag0014_genome. Supplemental material available at G3 online.
