# Supplementary material for: A haplotype-resolved reference genome for Eucalyptus grandis
Source: G3 (Bethesda). 2025 May 30;15(7):jkaf112. doi: 10.1093/g3journal/jkaf112 (PMC12239627; doi:10.1093/g3journal/jkaf112)
Supplement: jkaf112_Supplementary_Data [file jkaf112_supplementary_data.docx]

## A haplotype-resolved reference genome for *Eucalyptus grandis*

**Anneri Lötter^1^**, Tomas Bruna^2^, Tuan A. Duong^1^, Kerrie Barry^2^, Anna Lipzen^2^, Chris Daum^2^, Yuko Yoshinaga^2^, Jane Grimwood^3^, Jerry W. Jenkins^3^, Jayson Talag^4^, Justin Borevitz^5^, John T. Lovell^2,3^, Jeremy Schmutz^2,3^, Jill L. Wegrzyn^6^ and & Alexander A. Myburg^1,7,*^

^1^Department of Biochemistry, Genetics and Microbiology, Forestry and Agricultural Biotechnology Institute (FABI), University of Pretoria, Private bag X20, Pretoria 0028, South Africa

^2^Department of Energy Joint Genome Institute, Lawrence Berkeley National Laboratories, Mail Stop: 91R183, Berkeley, CA 94720, USA

^3^Genome Sequencing Center, HudsonAlpha Institute for Biotechnology, 601 Genome Way Northwest, Huntsville, AL 35806, USA

^4^Arizona Genomics Institute, University of Arizona, 1657 E. Helen St., Tucson, AZ 85721, USA

^5^Research School of Biology and Centre for Biodiversity Analysis, ARC Centre of Excellence in Plant Energy Biology, Australian National University, Canberra, ACT 0200, Australia

^6^Department of Ecology and Evolutionary Biology, Institute for Systems Genomics: Computational Biology Core, University of Connecticut, Storrs, CT 06269, USA

^7^Department of Genetics, Stellenbosch University, Private bag X1, Stellenbosch 7600, South Africa

*To whom correspondence should be addressed: zandermyburg@sun.ac.za

**Keywords:** *Eucalyptus grandis*, Genome improvement*,* Phased assembly, Tandem duplications,

### Supplementary figures


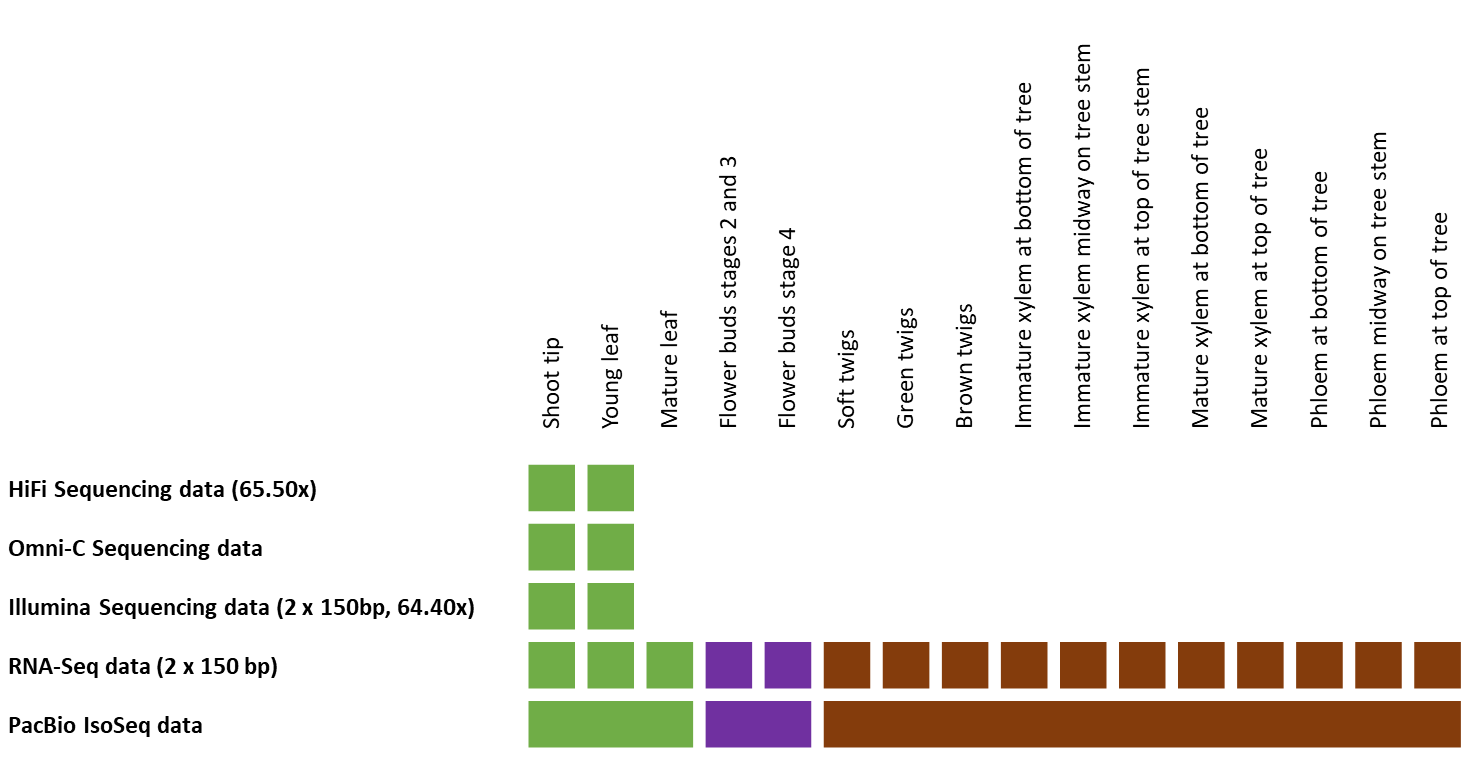


**Figure S1. Genome resources overview.** The source of the tissue used to generate the relevant sequencing data is indicated at the top. PacBio Iso-Seq sequencing data was generated in tissue pools consisting of the tissues indicated. The type of sequencing and approximate coverage is provided in brackets.


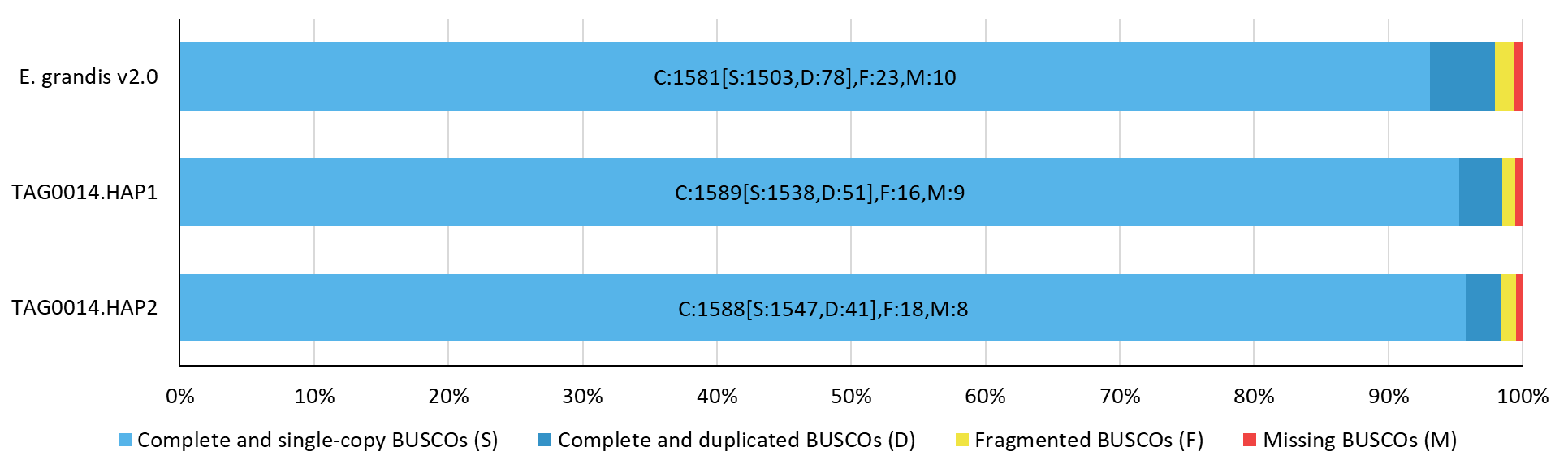


**Figure S2. BUSCO completion scores of reference genome assemblies.**

### Supplementary tables

**Table S1. Genome assembly and contiguity statistics.**

|  | **TAG0014 HAP1** | | **TAG0014 HAP2** | |
| --- | --- | --- | --- | --- |
| **QUAST assembly statistics** | | | | |
| # contigs (>= 0 bp) | 12 | | 11 | |
| # contigs (>= 1000 bp) | 12 | | 11 | |
| # contigs (>= 5000 bp) | 12 | | 11 | |
| # contigs (>= 10000 bp) | 12 | | 11 | |
| # contigs (>= 25000 bp) | 12 | | 11 | |
| # contigs (>= 50000 bp) | 12 | | 11 | |
| Total length (>= 0 bp) | 570,830,339 | | 552,407,773 | |
| Total length (>= 1000 bp) | 570,830,339 | | 552,407,773 | |
| Total length (>= 5000 bp) | 570,830,339 | | 552,407,773 | |
| Total length (>= 10000 bp) | 570,830,339 | | 552,407,773 | |
| Total length (>= 25000 bp) | 570,830,339 | | 552,407,773 | |
| Total length (>= 50000 bp) | 570,830,339 | | 552,407,773 | |
| # contigs | 12 | | 11 | |
| Largest contig | 68,844,007 | | 66,164,710 | |
| Total length | 570,830,339 | | 552,407,773 | |
| GC (%) | 39.50 | | 39.48 | |
| N50 | 57,174,188 | | 55,768,775 | |
| N90 | 37,932,624 | | 36,908,141 | |
| auN | 54,538,915 | | 52,725,103 | |
| L50 | 5 | | 5 | |
| L90 | 10 | | 10 | |
| # N's per 100 kbp | 43.80 | | 63.36 | |
|  |  |  |  |  |
| **Scaffolds are divided into the following sets** | | | | |
| **Set** | **Number of scaffolds** | **Size** | **Number of scaffolds** | **Size** |
| Main genome | 12 | 570.8 Mbp | 11 | 552.4 Mbp |
| Redundant (scaffolds composed of ≥95% 24mers >2x in all scaffolds) | 63 | 8.9 Mbp | 100 | 13.9 Mbp |
| Repetitive (≤250 kbp scaffolds composed of ≥95% 24mers >4x in ≥5 Mbp scaffolds) | 39 | 3.9 Mbp | 21 | 1.5 Mbp |
| Mitochondria (assembled using OatK) | 1 | 456.9 kbp | 1 | 456.9 kbp |
| Chloroplast (assembled using OatK) | 1 | 160.2 kbp | 1 | 160.2 kbp |
|  |  |  |  |  |
| **Chromosome statistics** | | | | |
| **Scaffold** | **Number of contigs** | **Scaffold Size** | **Number of contigs** | **Scaffold Size** |
| Chr01 | 5 | 44,764,794 | 4 | 41,459,349 |
| Chr02 | 6 | 57,174,188 | 4 | 56,979,195 |
| Chr03 | 3 | 64,190,686 | 4 | 61,673,418 |
| Chr04 | 3 | 37,932,624 | 2 | 36,908,141 |
| Chr05 | 2 | 67,041,112 | 2 | 64,932,641 |
| Chr06 | 4 | 55,026,902 | 9 | 53,665,386 |
| Chr07 | 5 | 58,892,594 | 4 | 55,768,775 |
| Chr08 | 3 | 68,844,007 | 3 | 66,164,710 |
| Chr09 | 2 | 36,944,981 | 6 | 36,522,489 |
| Chr10 | 1 | 38,023,239 | 3 | 37,128,649 |
| Chr11 | 2 | 41,944,707 | 5 | 41,205,020 |
| Remaining | 1 | 50,505 | 0 | 0 |
|  |  |  |  |  |
| **Assembly completeness notes** | | | | |
| **Total primary transcripts (34,121)** | **Number of sequences** | **Percentage of sequences** | **Number of sequences** | **Percentage of sequences** |
| Sequences placed at 90% identity and 85% coverage | 32,663 | 95.73 | 32,577 | 95.47 |
| Sequences aligned at <50% coverage | 1,068 | 3.13 | 1,171 | 3.43 |
| Sequences not found | 390 | 1.14 | 373 | 1.09 |
|  |  |  |  |  |
| **Merqury consensus quality (QV)** |  |  |  |  |
| **Assembly** | **Unique k-mers** | **Total k-mers** | **QV** | **Error rate** |
| TAG0014 HAP1 | 36,792 | 570,579,599 | 55.13 | 3.07E-06 |
| TAG0014 HAP2 | 30,892 | 552,056,853 | 55.74 | 2.66E-06 |
| Both | 67,684 | 1,122,636,452 | 55.42 | 2.87E-06 |
|  |  |  |  |  |
| **Merqury completeness** |  |  |  |  |
| **Assembly** | **k-mers used** | **k-mers in assembly** | **k-mers in reads** | **Completeness %** |
| TAG0014 HAP1 | all | 397,094,660 | 523,614,206 | 75.84 |
| TAG0014 HAP2 | all | 390,789,882 | 523,614,206 | 74.63 |
| Both | all | 514,083,312 | 523,614,206 | 98.18 |

**Table S2. BUSCO genome assembly and annotation completeness statistics for the *E. grandis* reference genomes.**

| **Genome** | ***E grandis* v2.0** | **TAG0014 HAP1** | **TAG0014 HAP2** |
| --- | --- | --- | --- |
| **Assembly BUSCO** | | | |
| **Percentages** | C:97.9% [S:93.1%, D:4.8%], F:1.4%, M:0.7% | C:98.5% [S:95.3%, D:3.2%], F:1.0%, M:0.5% | C:98.3% [S:95.8%, D:2.5%], F:1.1%, M:0.6% |
| **Complete BUSCOs (C)** | 1,581 | 1,589 | 1,588 |
| **Complete and single-copy BUSCOs (S)** | 1,503 | 1,538 | 1,547 |
| **Complete and duplicated BUSCOs (D)** | 78 | 51 | 41 |
| **Fragmented BUSCOs (F)** | 23 | 16 | 18 |
| **Missing BUSCOs (M)** | 10 | 9 | 8 |
| **Total BUSCO groups searched** | 1,614 | 1,614 | 1,614 |
|  | | | |
| **Annotation BUSCO** | | | |
| **Percentages** | C:93.8% [S:88.8%, D:5.0%], F:3.5%, M:2.7% | C:99.4% [S:96.2%, D:3.2%], F:0.4%, M:0.2% | C:99.5% [S:96.7%, D:2.8%], F:0.4%, M:0.1% |
| **Complete BUSCOs (C)** | 1,515 | 1,605 | 1,605 |
| **Complete and single-copy BUSCOs (S)** | 1,434 | 1,553 | 1,560 |
| **Complete and duplicated BUSCOs (D)** | 81 | 52 | 45 |
| **Fragmented BUSCOs (F)** | 56 | 6 | 6 |
| **Missing BUSCOs (M)** | 43 | 3 | 3 |
| **Total BUSCO groups searched** | 1,614 | 1,614 | 1,614 |

**Table S3. Number of heterozygous and homozygous sites fixed with polishing with Illumina (homozygous) and PacBio CCS (heterozygous) data.**

|  | **TAG0014 HAP1** | | **TAG0014 HAP2** | |
| --- | --- | --- | --- | --- |
| **SNP/INDEL Fixing stage** | Pre SNP/INDEL fixing | Post SNP/INDEL fixing | Pre SNP/INDEL fixing | Post SNP/INDEL fixing |
| **Heterozygous SNPs** | 5,298,407 | 5,298,497 | 5,245,389 | 5,245,433 |
| **Homozygous SNPs** | 95 | 27 | 148 | 30 |
| **Heterozygous INDELs** | 534,797 | 535,451 | 533,158 | 533,785 |
| **Homozygous INDELs** | 3,804 | 51 | 4,109 | 61 |
| **Callable Bases** | 546,533,710 | 546,535,795 | 531,387,543 | 531,395,648 |

**Table S4. Regions that were misphased and corrected as identified by Omni-C.**

| **Chromosome** | **Haplotype** | **Start** | **End** | **Haplotype** | **Start** | **End** |
| --- | --- | --- | --- | --- | --- | --- |
| Chr01 | HAP1 | 1,172,920 | 2,957,920 | HAP2 | 1 | 1,930,000 |
| Chr11 | HAP1 | 29,038,515 | 30,269,627 | HAP2 | 27,423,662 | 28,654,774 |

**Table S5. Repeat content of TAG0014 haplotype phased assemblies.**

|  | **TAG0014 HAP1** | | | | **TAG0014 HAP2** | | | |
| --- | --- | --- | --- | --- | --- | --- | --- | --- |
| **Repeat element type** | **Number elements** | **Length occupied (bp)** | **Percentage** | **Number elements** | | **Length occupied (bp)** | **Percentage** |  |
| DNA |  |  |  |  | |  |  |  |
| DNA/CMC-EnSpm | 323 | 70,462 | 0.01 | 300 | | 62,631 | 0.01 |  |
| DNA/Dada | 150 | 40,262 | 0.01 | 150 | | 40,737 | 0.01 |  |
| DNA/hAT-Ac | 1,406 | 781,621 | 0.14 | 1,332 | | 761,165 | 0.14 |  |
| DNA/hAT-Charlie | 734 | 280,916 | 0.05 | 752 | | 270,476 | 0.05 |  |
| DNA/hAT-Tag1 | 172 | 149,826 | 0.03 | 166 | | 136,735 | 0.02 |  |
| DNA/IS3EU | 113 | 25,206 | 0 | 107 | | 23,363 | 0 |  |
| DNA/MULE-MuDR | 1,888 | 1,689,454 | 0.3 | 1,737 | | 1,554,936 | 0.28 |  |
| DNA/PIF-Harbinger | 1,314 | 923,841 | 0.16 | 1,198 | | 831,417 | 0.15 |  |
| DNA/Zisupton | 148 | 69,478 | 0.01 | 108 | | 39,346 | 0.01 |  |
| LINE |  |  |  |  | |  |  |  |
| LINE/L1 | 13,885 | 10,472,401 | 1.83 | 12,822 | | 9,669,214 | 1.75 |  |
| LINE/L1-Tx1 | 112 | 49,967 | 0.01 | 111 | | 54,932 | 0.01 |  |
| LINE/RTE-BovB | 140 | 38,777 | 0.01 | 130 | | 36,556 | 0.01 |  |
| Low complexity | 35,126 | 1,628,973 | 0.29 | 30,767 | | 1,472,798 | 0.27 |  |
| LTR |  |  |  |  | |  |  |  |
| LTR/Caulimovirus | 2,706 | 3,727,706 | 0.65 | 2,737 | | 3,744,898 | 0.68 |  |
| LTR/Copia | 34,383 | 51,161,910 | 8.96 | 34,640 | | 50,735,113 | 9.18 |  |
| LTR/Gypsy | 19,476 | 20,457,810 | 3.58 | 17,985 | | 19,075,600 | 3.45 |  |
| LTR/Pao | 294 | 98,938 | 0.02 | 263 | | 91,931 | 0.02 |  |
| PLE |  |  |  |  | |  |  |  |
| PLE/Chlamys | 297 | 51,079 | 0.01 | 280 | | 47,867 | 0.01 |  |
| RC |  |  |  |  | |  |  |  |
| RC/Helitron | 1,028 | 214,807 | 0.04 | 933 | | 198,370 | 0.04 |  |
| Retroposon/L1-dep | 596 | 317,920 | 0.06 | 602 | | 307,680 | 0.06 |  |
| rRNA | 406 | 441,698 | 0.08 | 321 | | 225,857 | 0.04 |  |
| Simple repeat | 166,769 | 6,917,998 | 1.21 | 164,099 | | 6,793,321 | 1.23 |  |
| SINE | 145 | 31,560 | 0.01 | 126 | | 22,117 | 0 |  |
| SINE/tRNA | 74 | 10,192 | 0 | 78 | | 10,471 | 0 |  |
| SINE/U | 122 | 34,019 | 0.01 | 118 | | 31,286 | 0.01 |  |
| snRNA | 66 | 8,046 | 0 | 58 | | 7,149 | 0 |  |
| tRNA | 312 | 26,683 | 0 | 313 | | 28,307 | 0.01 |  |
| Unknown | 461,811 | 157,020,984 | 27.51 | 439,724 | | 145,479,922 | 26.34 |  |
| **Total** | **743,996** | **256,742,534** | **44.99** | **711,957** | | **241,754,195** | **43.78** |  |

**Table S6. Gene annotation statistics for the TAG0014 HAP1 and HAP2 reference genome assemblies.**

|  | **TAG0014 HAP1** | **TAG0014 HAP2** |
| --- | --- | --- |
| Primary transcripts (loci) | 35,929 | 35,583 |
| Alternative transcripts | 28,647 | 28,368 |
| Total transcripts | 64,576 | 63,951 |
|  |  |  |
| For primary transcripts: |  |  |
| Average number of exons | 5.1 | 5.0 |
| Median exon length | 179 | 179 |
| Median intron length | 208 | 209 |
|  |  |  |
| **Gene model support (value is number of gene models):** | | |
| Any EST support | 30,914 | 30,666 |
| EST support over 100% of their lengths | 29,269 | 29,071 |
| EST support over 95% of their lengths | 29,526 | 29,315 |
| EST support over 90% of their lengths | 29,666 | 29,450 |
| EST support over 75% of their lengths | 29,954 | 29,710 |
| EST support over 50% of their lengths | 30,255 | 30,000 |
| Peptide homology coverage of 100% | 7,623 | 7,551 |
| Peptide homology coverage of over 95% | 25,444 | 25,314 |
| Peptide homology coverage of over 90% | 28,062 | 27,873 |
| Peptide homology coverage of over 75% | 31,129 | 30,789 |
| Peptide homology coverage of over 50% | 32,879 | 32,606 |
| Pfam annotation | 28,139 | 27,845 |
| Panther annotation | 32,935 | 32,563 |
| KOG annotation | 15,954 | 15,781 |
| KEGG Orthology annotation | 13,671 | 13,636 |
| E.C. number annotation | 11,333 | 11,271 |

**Table S7. gFACS summary statistics of *E. grandis* v2.0 reference genome and TAG0014 haplotype phased assemblies.**

|  | ***E. grandis* v2.0** | **TAG0014 HAP1** | **TAG0014 HAP2** |
| --- | --- | --- | --- |
| Number of genes | 36,349 | 35,929 | 35,583 |
| Number of monoexonic genes | 7,611 | 6,182 | 6,165 |
| Number of multiexonic genes | 28,738 | 29,747 | 29,418 |
|  | | | |
| Number of positive strand genes | 18,244 | 18,064 | 17,694 |
| Monoexonic | 3,935 | 3,121 | 3,093 |
| Multiexonic | 14,309 | 14,943 | 14,601 |
| Number of negative strand genes | 18,105 | 17,865 | 17,889 |
| Monoexonic | 3,676 | 3,061 | 3,072 |
| Multiexonic | 14,429 | 14,804 | 14,817 |
|  |  |  |  |
| Average overall gene size | 3,104.21 | 4,020.55 | 3,994.77 |
| Median overall gene size | 2,402 | 3,083 | 3,071 |
| Average overall CDS size | 2,000.79 | 3,842.28 | 3,827.66 |
| Median overall CDS size | 1,394.00 | 1,974.50 | 1,971.00 |
| Average overall exon size | 287.21 | 347.69 | 347.74 |
| Median overall exon size | 156 | 163 | 163 |
|  |  |  |  |
| Average size of monoexonic genes | 911.57 | 1,146.96 | 1,139.47 |
| Median size of monoexonic genes | 676 | 933.00 | 931.50 |
| Largest monoexonic gene | 5,377 | 7,502 | 10,192 |
| Smallest monoexonic gene | 201 | 105 | 99 |
|  |  |  |  |
| Average size of multiexonic genes | 3,684.90 | 4,617.74 | 4,593.14 |
| Median size of multiexonic genes | 3,010 | 3,627 | 3,621 |
| Largest multiexonic gene | 56,415 | 92,530 | 113,073 |
| Smallest multiexonic gene | 212 | 135 | 199 |
|  |  |  |  |
| Average size of multiexonic CDS | 2,289.26 | 4,402.42 | 4,391.01 |
| Median size of multiexonic CDS | 1,617 | 2,312 | 2,315 |
| Largest multiexonic CDS | 43,282 | 402,181 | 400,345 |
| Smallest multiexonic CDS | 201 | 105 | 96 |
|  |  |  |  |
| Average size of multiexonic exons | 267.86 | 335.05 | 335.08 |
| Median size of multiexonic exons | 151 | 160 | 160 |
| Average size of multiexonic introns | 344.60 | 338.49 | 337.84 |
| Median size of multiexonic introns | 147 | 125 | 125 |
|  |  |  |  |
| Average number of exons per multiexonic gene | 8.55 | 13.14 | 13.11 |
| Median number of exons per multiexonic gene | 5 | 6 | 6 |
| Largest multiexonic exon | 7,890 | 10,064 | 13,880 |
| Smallest multiexonic exon | 1 | 1 | 1 |
| Most exons in one gene | 301 | 1,013 | 1,010 |
|  |  |  |  |
| Average number of introns per multiexonic gene | 7.55 | 12.21 | 12.18 |
| Median number of introns per multiexonic gene | 4 | 5 | 5 |
| Largest intron | 9,190 | 87,554 | 39,127 |
| Smallest intron | -1 | -1 | -1 |
|  |  |  |  |
| **The following columns do not involve codons** |  |  |  |
| Number of complete models | 36,098 | 35,866 | 35,523 |
| Number of 5' only incomplete models | 70 | 28 | 28 |
| Number of 3' only incomplete models | 181 | 20 | 14 |
| Number of 5' and 3' incomplete models | 0 | 15 | 18 |

**Table S8. Summary of structural variants between the reference genomes.**

| **Reference genome** | **Query genome** | **Number of events** | **Length reference (bp)** | **Percentage reference (%)** | **Length query (bp)** | **Percentage query (%)** |
| --- | --- | --- | --- | --- | --- | --- |
| **Syntenic regions** | | | | | | |
| *E. grandis* v2.0 | TAG0014 HAP1 | 14,029 | 369,254,214 | 53.41 | 370,195,762 | 64.85 |
| TAG0014 HAP1 | TAG0014 HAP2 | 13,349 | 396,000,960 | 69.37 | 396,333,150 | 71.75 |
| TAG0014 HAP2 | *E. grandis* v2.0 | 14,285 | 371,533,224 | 67.26 | 370,664,106 | 53.61 |
| **Inversions** | | | | | | |
| *E. grandis* v2.0 | TAG0014 HAP1 | 100 | 18,352,964 | 2.65 | 16,783,397 | 2.94 |
| TAG0014 HAP1 | TAG0014 HAP2 | 89 | 9,417,568 | 1.65 | 8,282,210 | 1.5 |
| TAG0014 HAP2 | *E. grandis* v2.0 | 114 | 7,753,224 | 1.4 | 7,569,510 | 1.09 |
| **Translocations** | | | | | | |
| *E. grandis* v2.0 | TAG0014 HAP1 | 9,982 | 72,043,085 | 10.42 | 72,102,959 | 12.63 |
| TAG0014 HAP1 | TAG0014 HAP2 | 7,827 | 46,538,476 | 8.15 | 46,282,393 | 8.38 |
| TAG0014 HAP2 | *E. grandis* v2.0 | 10,090 | 76,720,961 | 13.89 | 75,412,281 | 10.91 |
| **Duplications - reference** | | | | | | |
| *E. grandis* v2.0 | TAG0014 HAP1 | 22,713 | 84,524,332 | 12.23 |  |  |
| TAG0014 HAP1 | TAG0014 HAP2 | 21,289 | 82,095,122 | 14.38 |  |  |
| TAG0014 HAP2 | *E. grandis* v2.0 | 19,387 | 73,653,973 | 13.33 |  |  |
| **Duplications - query** | | | | | | |
| *E. grandis* v2.0 | TAG0014 HAP1 | 17,683 |  |  | 56,211,493 | 9.85 |
| TAG0014 HAP1 | TAG0014 HAP2 | 14,659 |  |  | 48,917,664 | 8.86 |
| TAG0014 HAP2 | *E. grandis* v2.0 | 19,526 |  |  | 58,631,124 | 8.48 |
| **Not aligned - reference** | | | | | | |
| *E. grandis* v2.0 | TAG0014 HAP1 | 26,566 | 107,699,067 | 15.58 |  |  |
| TAG0014 HAP1 | TAG0014 HAP2 | 20,602 | 75,546,270 | 13.23 |  |  |
| TAG0014 HAP2 | *E. grandis* v2.0 | 21,454 | 76,972,257 | 13.93 |  |  |
| **Not aligned - query** | | | | | | |
| *E. grandis* v2.0 | TAG0014 HAP1 | 22,602 |  |  | 83,357,649 | 14.6 |
| TAG0014 HAP1 | TAG0014 HAP2 | 18,917 |  |  | 65,828,782 | 11.92 |
| TAG0014 HAP2 | *E. grandis* v2.0 | 27,446 |  |  | 114,939,770 | 16.63 |

**Table S9. Summary of local variants between the reference genomes.**

| **Reference genome** | **Query genome** | **Number of events** | **Length reference (bp)** | **Length query (bp)** |
| --- | --- | --- | --- | --- |
| **Single nucleotide polymorphisms (SNPs)** | | | | |
| *E. grandis* v2.0 | TAG0014 HAP1 | 6,453,759 | 6,453,759 | 6,453,759 |
| TAG0014 HAP1 | TAG0014 HAP2 | 6,197,893 | 6,197,893 | 6,197,893 |
| TAG0014 HAP2 | *E. grandis* v2.0 | 6,675,143 | 6,675,143 | 6,675,143 |
| **Insertions** | | | | |
| *E. grandis* v2.0 | TAG0014 HAP1 | 696,627 |  | 16,783,397 |
| TAG0014 HAP1 | TAG0014 HAP2 | 550,834 |  | 8,282,210 |
| TAG0014 HAP2 | *E. grandis* v2.0 | 626,069 |  | 7,569,510 |
| **Deletions** | | | | |
| *E. grandis* v2.0 | TAG0014 HAP1 | 494,858 | 4,448,538 |  |
| TAG0014 HAP1 | TAG0014 HAP2 | 482,009 | 4,267,853 |  |
| TAG0014 HAP2 | *E. grandis* v2.0 | 592,575 | 5,487,780 |  |
| **Copygains (CPG)** | | | | |
| *E. grandis* v2.0 | TAG0014 HAP1 | 1,626 |  | 5,703,905 |
| TAG0014 HAP1 | TAG0014 HAP2 | 1,274 |  | 3,698,389 |
| TAG0014 HAP2 | *E. grandis* v2.0 | 1,406 |  | 4,562,277 |
| **Copylosses (CPL)** | | | | |
| *E. grandis* v2.0 | TAG0014 HAP1 | 1,426 | 4,868,625 |  |
| TAG0014 HAP1 | TAG0014 HAP2 | 1,219 | 3,916,910 |  |
| TAG0014 HAP2 | *E. grandis* v2.0 | 1,639 | 5,566,419 |  |
| **Highly diverged regions (HDR)** | | | | |
| *E. grandis* v2.0 | TAG0014 HAP1 | 9,254 | 29,765,501 | 27,608,817 |
| TAG0014 HAP1 | TAG0014 HAP2 | 4,849 | 16,182,833 | 15,199,906 |
| TAG0014 HAP2 | *E. grandis* v2.0 | 9,347 | 25,316,307 | 25,061,446 |
| **Tandem repeats (TDM)** | | | | |
| *E. grandis* v2.0 | TAG0014 HAP1 | 312 | 549,240 | 659,116 |
| TAG0014 HAP1 | TAG0014 HAP2 | 286 | 970,007 | 1,121,248 |
| TAG0014 HAP2 | *E. grandis* v2.0 | 328 | 926,751 | 687,677 |

**Table S10. Number of tandem genes and tandem arrays in the reference genomes.**

|  | ***E. grandis* v2.0** | **TAG0014 HAP1** | **TAG0014 HAP2** |
| --- | --- | --- | --- |
| Number of genes | 5,488 | 6,493 | 6,346 |
| Number of arrays | 2,106 | 2,235 | 2,232 |
| Mean number of genes | 2.62 | 2.91 | 2.84 |
| Minimum number of genes within an array | 2 | 2 | 2 |
| Maximum number of genes within an array | 34 | 33 | 36 |
| Proportion of annotated genes (%) | 15.10 | 18.40 | 17.83 |
